# Nosocomial Infection Outbreak due to SARS-COV-2 in a Hospital Unit of Particularly Vulnerable Patients

**DOI:** 10.7150/ijms.53270

**Published:** 2021-03-19

**Authors:** Rocío Seijo Bestilleiro, Diana Martinez Señaris, María José Pereira Rodríguez, Rita Galeiras Vázquez, Raquel García Rodríguez, María Teresa García Rodriguez, Cristina González Martín, María Teresa Seoane Pillado, Vanesa Balboa Barreiro, Valentín Valdés Valiña, Sonia Pértega Díaz

**Affiliations:** 1 Research group in Nursing and Health Care, Instituto de Investigación Biomédica de A Coruña (INIBIC), Complexo Hospitalario Universitario de A Coruña (CHUAC), Sergas, Universidade da Coruña, A Coruña, Spain.; 2 Haematology and Haemotherapy Department, Complexo Hospitalario Universitario de A Coruña (CHUAC), Sergas, A Coruña, Spain.; 3 Preventive Medicine Department, Complexo Hospitalario Universitario de A Coruña (CHUAC), Sergas, A Coruña, Spain.; 4 Intensive Care Unit, Complexo Hospitalario Universitario de A Coruña (CHUAC), Sergas, A Coruña, Spain.; 5 Research group in Rheumatology and Health, Universidade da Coruña, A Coruña, Spain.

**Keywords:** COVID-19, Coronavirus Infections, Disease outbreaks, Health personnel, Infectious Disesase Transmission, Patient-to-Professional, Infectious Disease Transmission, Professional-to-Patient.

## Abstract

**Objectives:** To report a COVID-19 outbreak among workers and inpatients at a medical ward for especially vulnerable patients.

**Methods:** Descriptive study of a nosocomial COVID-19 outbreak registered in March-April 2020 at medical ward of onco-hematological patients in an Spanish hospital. Confirmed cases were hospitalized patients, healthcare and non-healthcare workers who tested positive by PCR on a nasopharyngeal swab.

**Results:** Twenty-two COVID-19 cases (12 workers and 10 inpatients) were laboratory-confirmed. Initial cases were a healthcare provider and a visitor who tested positive. The median patients age was 73 years (range 62-88). The main reason of admission was haematological in 8 patients and oncologic in 2. All patients followed an immunosuppressive treatment, 5/10 with high-flow oxygen nebulizations. Five patients presented a moderate/serious evolution, and 5 patients died. The mean workers age was 42.1±10.9. One healthworker required Intensive Care Unit admission, and all of them recovered completely.

**Conclusions:** In the hospital setting, close patients surveillance for SARS-CoV-2 is essential, especially in immunosuppressed patients. Replacing nebulizations or high-flow oxygen therapies, when other equivalent options were available, to reduce dispersion, and controlling ventilation ducts, together with hygiene measures and an active follow-up on inpatients, visitors and workers appear to be important in preventing nosocomial outbreaks.

## Introduction

The disease produced by SARS-CoV-2 (COVID-19) was identified for the first time in Wuhan, China, in December 2019 1, 2. It expanded rapidly, and it was declared a pandemic on 11 March 2020 3, 4, with identified cases in 213 countries 3.

In Spain, the first death took place in The Valencian Community on 13 February 2020. On 13 March 2020, the number of confirmed cases in Spain was 4,209, with 120 deaths 5. On 30 April 2020, the number of confirmed cases in Spain was 213,435, with 24,543 deaths 6.

The first case in Galicia (an autonomous region in the northwest of Spain) was identified on 4 March at the A Coruña University Hospital. On 13 March, a state of health emergency was declared in the Galician territory 7.

The clinical spectrum of the infection caused by SARS-CoV-2 varies from asymptomatic, mild respiratory symptoms with fever and dry cough, to acute respiratory distress syndrome (ARDS), multi-organic dysfunction, and death 8-9. Several studies have recorded between 50-60% of asymptomatic patients 10. In those individuals who have symptoms, fever has been detected in 43-99% of patients (>37.3 ºC) and cough in 70-80% of them 11-15.

Healthcare and non-healthcare workers who take care of non-COVID-19 patients have protection protocols and, a priori, less risk of infection. Patients placed in restricted units of non-COVID-19 pathologies are also provided with these protocols. However, the epidemiological surveillance services should be diligent in order to track signs and factors that could determine the presence of an outbreak, which would have fatal consequences in vulnerable people.

Nosocomial outbreaks in different health centres have been described in several parts of the world 10, 16-17. Several studies evidence the susceptibility of healthcare and non-healthcare workers to be infected, especially after being exposed to patients initially not suspected of COVID-19, who are likely to transmit the virus at the pre-symptomatic or asymptomatic stages 18-23. The fact that sometimes this disease is present almost without symptoms could mean a bigger risk in non-COVID-19 units.

This study reports an outbreak of COVID-19 among healthcare workers, non-healthcare workers and inpatients at a unit of haematological and internal medicine patients in a tertiary referral Spanish hospital. The aim of the investigation is to explore the causes and characteristics of the outbreak, as well as the measures implemented in order to prevent and control it. The results may suggest factors that can contribute to the propagation of the infection in hospitals, in order to minimize the infections in these centres in the future.

## Material and Methods

### Outbreak setting

The outbreak occurred in March and April 2020, at a medical ward in A Coruña University Hospital. This is a 1348-bed tertiary hospital serving a population of 501,526 inhabitants in the Northwest of Spain.

The contingency plan implemented during the pandemic followed the national and international recommendations. Thus, care units for patients with COVID-19 were created, with well-defined circuits from the admission, with the objective of minimizing risks both for patients and for workers.

The Unit where the outbreak was registered has 34 beds that, according to the hospital reorganization during the pandemic, was destined for patients with acute pathologies (non-COVID-19) that required the assistance of Haematologist, Oncologist, or Internal Medicine doctors. According to the hospital protocol, during this period only the visit of one relative was allowed (under 70 years old and without symptoms) per patient.

**Figure 1:**
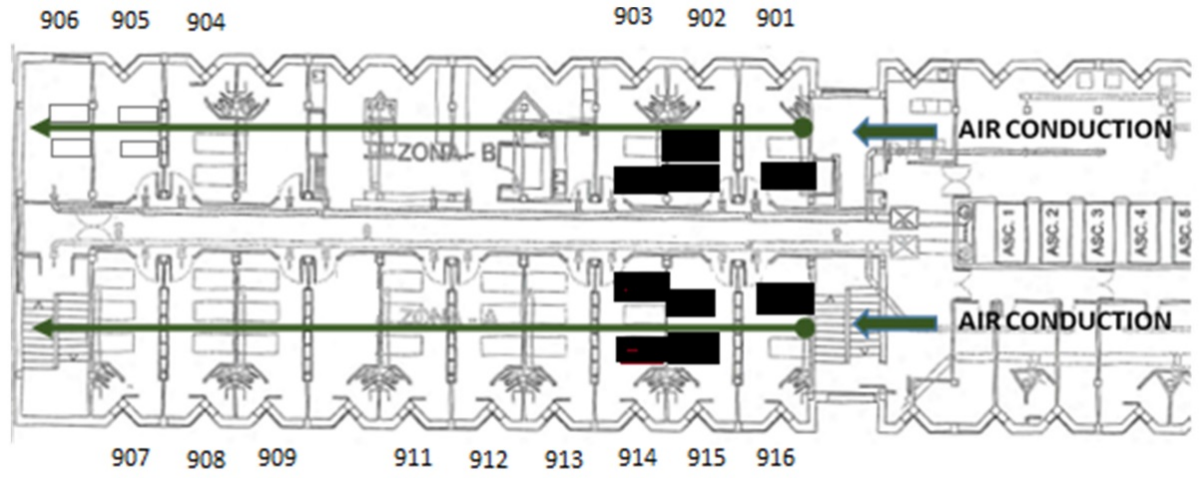
Distribution of the Unit, entrance, and direction of the airflow. The beds of the infected patients are highlighted in bold.

The ward comprises an area of 368 m^2^, with 15 double or triple rooms and 1 nursing station, 1 doctor's office, 1 security control y 2 storage rooms. There is a single point of entry without direct access to other units. The floor plan of the hospital ward is shown in Figure 1. When the first case was confirmed, 34 out of the 34 beds of the ward were occupied. The air passage in the unit goes through an independent channel on each side, located at the entrance of the unit (Figure 1). The airflow goes from the entrance to the end of the floor through inside of all of the rooms. This is not a unit that is actively ventilated due to the characteristics of immunosuppression of its patients (especially rooms 901, 902, 903, 915, and 916).

The Unit team has 28 healthcare workers (4 doctors: 2 internists, 1 haematologist, and 1 oncologist, 14 nurses, 10 nursing assistants), and non-healthcare workers (1 cleaner). During the pandemic, both workers and patients were required to wear a surgical mask, to keep the interpersonal distance whenever possible, and to wash their hands before and after any contact with patients, devices or their surroundings. The healthcare professionals were informed of the need to communicate the centre if they had a fever or any other symptoms compatible with this infection. The centre had a hotline for workers to solve doubts and to inform of sick leave in case of not coming to the hospital.

### Case definitions and identification

The population examined were inpatients, healthcare workers (doctors, nurses, nursing assistants) and non-healthcare workers (cleaning staff) of the unit.

The confirmed cases are defined as inpatients, healthcare or non-healthcare workers with a positive Polymerase Chain Reaction (PCR) test for SARS-CoV-2 in the analysis of a nasopharyngeal sample obtained through a swab. Samples were taken to the Microbiology service both for inpatients and for outpatients. As for the latter, the samples were obtained in a dedicated testing site next to the hospital, named *Auto-COVID* (the patient arrives in their car and they are tested without getting out). In both cases, a nurse carried out the test, according to the protocol and procedure of the hospital.

### Measurements

This study was authorized by the Hospital General Management in compliance with national and regional regulations. Demographic characteristics were registered for each case, as well as previous comorbidities. In addition, for infected patients, their diagnosis, treatment with immunosuppressants or not, high-flow oxygen nebulizations, active concomitant infections, and ECOG scale of performance status were also recorded 24. Laboratory studies and X-ray images were not registered.

In accordance with the patients' symptoms, the seriousness of the disease caused by SARS-CoV-2 was divided into four classes: mild, moderate, severe, and critical 25-27. We also add the “asymptomatic” category to our study.

If patients have slight clinical symptoms without pneumonia or hypoxia they are treated as having a mild condition. When patients have clinical signs of pneumonia (fever, cough, dyspnoea, fast breathing, fingertip blood oxygen saturation >90%) and do not fit severe or critical group, they are identified as having a moderate condition. Severe condition applies when patients experience respiratory distress and a respiratory rate of >30 times per minute and a fingertip blood oxygen saturation of <90% at rest. Patients were regarded as having a critical condition if they had any of the following: respiratory failure requiring mechanical ventilation, shock, and other organ failures requiring ICU treatment 25-27. In-hospital deaths were recorded.

### Outbreak description and control measures

**Figure 2:**
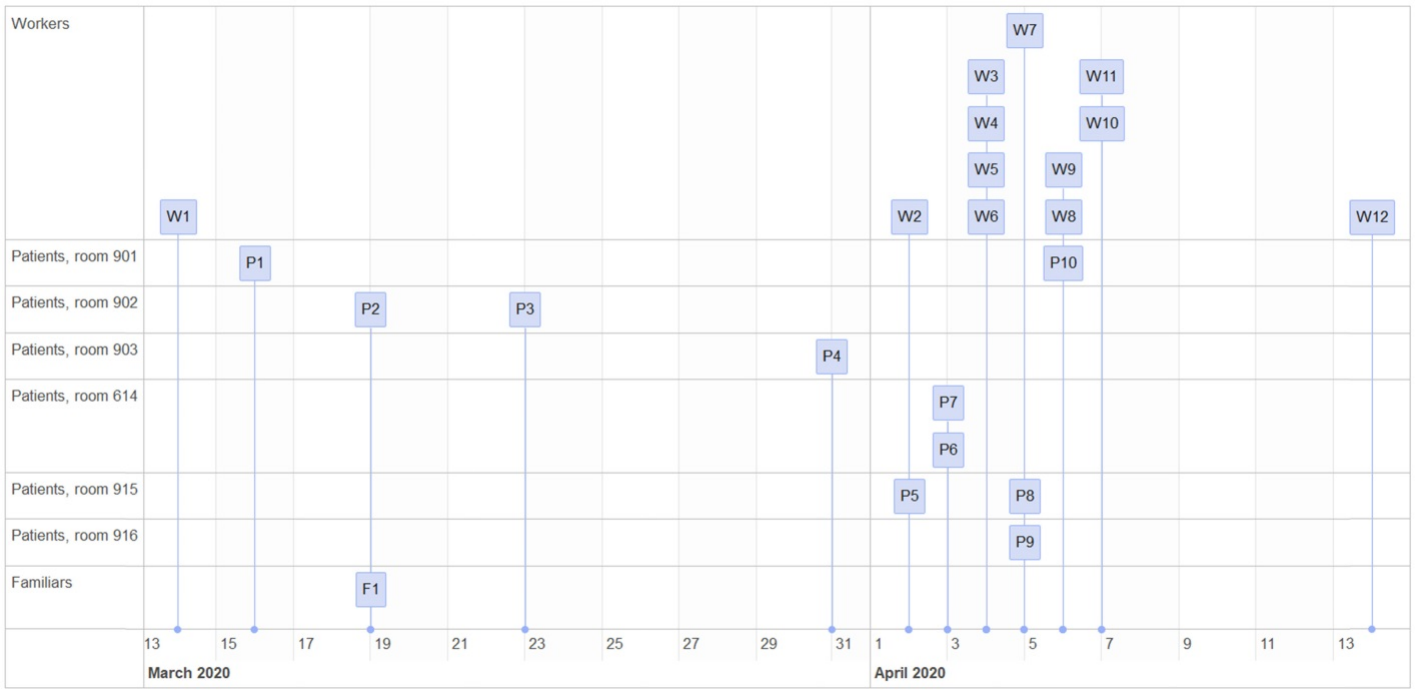
Diagnosis of cases in patients and workers.

The case detection sequence is represented in Figure 2. Tables 1 and 2 show the characteristics of the patients and workers affected, respectively, along with their evolution.

On 13 March 2020, the Department of Preventive Medicine was informed of a potential case of infection by SARS-CoV 2 in a healthcare worker of the Haematology-Internal Medicine Unit, who showed non-productive cough (HCW 1). One co-habitant of this worker, who had travelled to a risk area on the East coast of Spain in which the number of cases had been increasing, presented identical symptomatology a week before. The first day HCW 1 experienced symptoms (13 March), the Department of Preventive Medicine was notified. He was quarantined at home until the performance of the PCR test. A contact tracing in the unit was not performed at that point since it was considered that the infectiousness of the disease started in the symptomatic stage, and HCW 1 had followed the hospital instructions, had worked with a surgical mask, and had not presented any previous symptoms 28.

HCW 1 had worked in rooms 901 and 902 during the morning shift of the 11 and 12 March. The test was carried out on 14 March, which was positive for SARS-CoV-2, as well as the test carried out to his co-habitant on 15 March, which was also positive. In the following days, new cases in patients and companions who had been in rooms 901 and 902 were identified.

The first case in inpatients was detected in a Haematology patient with immunosuppressive treatment (PCR positive on 16 March) who was in room 901 and was discharged on 13 March. Later, on 16 March, she was admitted to another unit with compatible symptomatology with COVID-19. Since the patient did not re-enter the same unit and she was treated by the worker in the pre-symptomatic stage of the disease, an epidemiologic link was not initially established.

Patients 2 (PCR positive on 19 March) and 3 (PCR positive on 23 March) also suffered hemopathies and they shared room 902. A PCR test for SARS-CoV-2 was requested for patient 2 due to compatible symptoms. Her companion, after the diagnosis, informed of having experienced symptoms (fever and cough) for several days when he attended the Hospital as a companion. He explained that he did not report it in order not to separate from their relative, who had a very unfavourable prognosis with foreseeable death. He was tested on the same day as the patient, with a positive result. Patient 2 was rapidly progressing their disease and had previous comorbidities. On the day of the diagnosis and before knowing the companion's situation, the patient received high-flow oxygen therapy and bronchodilators nebulized due to acute respiratory deterioration. It is worth to highlight that on the same day when patient 3 was examined as a close contact of patient 2, she initially tested negative (PCR on 19 March). Since there were no symptoms, and having into account complications and previous comorbidities, isolating was interrupted. However, she experienced fever after 3 days, and the test result was positive. In this case, the patient underwent a high-flow oxygen therapy as well as nebulized bronchodilators for practically her whole stay. After the results, all patients that tested positive were referred to a COVID-19 unit.

Patient 4 (room 903), also an haematology patient, tested positive by PCR on 31 March. She suffered from a disease with unfavourable short-term prognosis and was following an immunosuppressive treatment, in addition to other serious complications. She had experienced fever on 28 March, but that was linked to their complications and basal disease. However, after 3 days the fever reappeared, and although she had not had, *a priori*, contact with any known positive cases nor did she show any deteriorated respiratory condition, the PCR test was positive. The patient undertook low-flow oxygen therapy via nasal cannulas.

On 2 April 2020, both a nurse (HCW 2) and a patient (Patient 5, room 915) tested positive by PCR. The patient was haematological and was following an immunosuppressive treatment, and was tested because of fever without another symptomatology. HCW 2 had fever for 3 days and was isolated at home since the beginning of the symptoms.

After these results, a screening of the patients who were in the Unit was carried out. On 3 April, patient 6 and 7 (both in room 914) tested positive by PCR. These were two patients with cancer at a very advanced stage of their disease and other comorbidities.

In addition, a screening of all the workers of the floor started, obtaining positive results in 4 workers on 4 April (3 nurses and 1 cleaner). When a survey was carried out, workers who tested positive informed that they had only experienced anosmia. The rest of the asymptomatic workers who had been in contact with these patients were isolated at home and a PCR test was scheduled for 7 days after the risk contact.

On 5 April, patients 8 and 9 tested positive by PCR (rooms 915 and 916 respectively), both with haematological pathologies, following an immunosuppressive treatment, and both presented fever; as well as worker 7. Patient 8 tested negative on the test carried out on the same day as their roommate, who had tested positive (patient 5). Patient 9 had several negative previous tests but suffered from acute respiratory pathology through all their stay, which is why he was following treatment with aerosol-generating respiratory techniques, including non-invasive mechanical ventilation (NIMV). HCW 7 experienced general discomfort from the day before.

The last patient who tested positive in this outbreak was patient 10 (PCR positive on 6 April), room 901, who experienced fever and had also tested negative previously.

The rest of the staff who tested positive by PCR were 3 nurses (one on 6 April and two on 7 April) and 2 nursing assistants (on 6 April and 14 April). They were all isolated at home from the start of the screening (3 April). Both nurses and assistants experienced fever a day before the diagnosis.

## Results

Between 14 March and 15 April 2020, the total number of positive PCR tests for SARS-CoV-2 in this outbreak was 22 (12 workers y 10 inpatients). The first identified cases were HCW 1 and patient's 2 companion.

The number of patients who tested positive by PCR was 10 (4 women and 6 men). During the period when the nosocomial outbreak was identified, 60 patients were admitted to the unit, which shows a cumulative incidence of 16.7%. The average patient age was 72.9±8.0 years old (range: 62-88). The reason for admission was haematological in 8 patients and cancer conditions in other 2. Seven out of 10 patients were ECOG 3 (n=2) or 4 (n=5). All patients followed an immunosuppressive treatment, 5/10 with high-flow oxygen nebulizations and 8/10 presented a concomitant active infection.

Out of the total number of positive patients, 5 (50%) presented a moderate or serious evolution, without needing ICU admission. The other 5 patients died (3 haematological (37.5%) and 2 with cancer (100%)). Death occurred in patients with a very unfavourable evolution of their haematological diseases or cancer, with an estimated prognosis of less than 3 months.

The number of positive workers was 12 (9 women and 3 men), with an average age of 42.1±10.9 years old (range: 28-62 years old). The distribution of professional categories of affected workers was as follows: 7 nurses, 3 nursing assistants, and 1 cleaner. With regards to the evolution of the workers' disease, HCW 1 required admission to the ICU for 32 days (critical) and a nurse needed hospital admission (moderate), both presented previous pathologies (Table 2). The rest of the workers did not require admission, experiencing mild or moderate symptoms. None of the workers died and they all recovered completely.

The distribution of the positive cases in the unit was well-defined (rooms 901, 902, 903, 914, 915, and 916), all of them were located in the first rooms, close to the entrance of the unit, where the airflow through two parallel impellers began.

Five patients with positive PCR tests were treated with procedures that generated aerosols at the start of the outbreak (patient's numbers 2, 3, 6, 7, and 9). There were no positive patients of COVID-19 in any of the rooms of the centre nor the end of the unit. The last case was diagnosed on 14 April, a worker, a month after the first case.

## Discussion

This study shows the complexity of the approach of a nosocomial infection outbreak by SARS-CoV-2 and its impact, particularly in an area of vulnerable patients. The analysis reveals that the hospital areas designated to non-COVID-19 patients can be a high-risk environment for a SARS-CoV-2 outbreak.

Workers or companions, who interact in environments inside and outside the hospital, can be the start of the outbreak, although infected patients contribute to the diffusion of pathogens, and it is key to reduce or to prevent the spread of the infectious agent from its source.

Therefore, staff working in non-COVID-19 admissions areas follow protocols adapted to low-risk patients. This includes surgical masks (for workers and patients), adequate hand hygiene, and keeping a distance of at least 1.5 meters whenever possible, as well as to know and notify any symptoms that could suggest the disease by SARS-CoV-2. However, even in groups trained in preventive measures and with a high level of compliance, these recommendations may not be enough when they are exposed to a focus, particularly when the focus is initially unknown. In our case, the initial transmission from the very first case of the unit during the pre-symptomatic phase was identified (HCW1). The spread was also identified afterwards between patients and workers, contributing to the fast dissemination of the virus through the Unit, which has been described in other healthcare centres 19-21. On this matter, infection control and dissemination strategies based on monitoring after the start of symptoms, seemed insufficient to prevent the transmission, and especially if a case was already identified 10.

In this outbreak, the scale of the problem included half of the workers of the unit, with a clinical favourable evolution in most of them, except in one that required ICU admission.

Half of the patients who suffered from the outbreak died due to their disease and other comorbidities, not receiving Intensive Care Unit assistance.

Restricting relatives' visits in an epidemic is part of the infection control strategies. However, in the case of terminally-ill patients, the decision of the families to remain with them until the very end can lead them to hide their symptoms or a known infection, putting into risk a group of people.

These threats should be fought against with adequate information, individual responsibility, and actions directed from the alert and epidemiologic tracking services. As of the epidemiological surveillance, the strategy developed by the alert and epidemiologic tracking services seemed inefficient since it did not consider the asymptomatic phase like a period of risk, and did not have a system which allowed to carry out the traceability of cases between patients and workers promptly.

On the other hand, 50% of patients with SARS-Cov-2 disease received medical treatment with aerosol-generating techniques. Transmission through aerosols is biologically possible, and thus it has been shown in different studies 18. Real suspension times in the environment can increase when there are significant crossed flows, which is often the case in medical care environments 27. For example, doors that open, teams and beds moving around, people going up and down constantly, although we have not seen that the evolution of the outbreak followed the direction of the airflows of the Unit.

Joshua L. Santarpia et al. 29 present 13 individuals with COVID-19 in the University of Nebraska Medical Center. They took air and surface samples and they detected viral contamination in all the samples, which led them to conclude that SARS-CoV-2 could spread both through direct mechanisms (little drops and person to person) and indirect (contaminated objects and air transmission). In another study carried 30, high concentrations of the virus were found in poorly ventilated areas and in the absence of negative pressure. In the ICUs, with adequate ventilation and negative pressure, the load was lower. This was also the case in the areas designated to changing the PPE (Personal Protective Equipment), suggesting that movement could reintroduce in the airflow particles adhered to the PPE.

On this matter, surgical masks are designed to block the big particles, drops, and aerosols, but they are less efficient to block aerosols of small particles (less than 5 μm). That's why healthcare workers that carry out aerosol-generation procedures in patients with SARS-CoV-2 are required to wear other protective equipment, in accordance with the recommendations public health organisations 31, 32. In addition, there is epidemiologic data that proves that aerosol-generation procedures increase the health risk for workers during the SARS epidemic.

However, in the meta-analysis of Tran K, et al. 33 that studies the evidence of acute respiratory infections transmission risk in healthcare workers, the transmission risk associated to the nebulizing treatment was not statistically significant (two cohort studies; pooled OR 3.7; 95% CI 0.7, 19.5). These findings must be interpreted in the context of the very low quality of the studies.

We thought that, in the outbreak described, it could not be discarded the possibility that the generation of aerosols could have influenced the spread of the virus. Future researches could help identify places and practices of work in which the transmission via aerosols is more likely to occur, so exposition risks and controls can be more efficient 34. Nowadays, recommendations for prevention and infection control related to healthcare assistance in a pandemic period, suggest avoiding the use of techniques that generate aerosols, if there are therapeutic alternatives.

Patients that present onco-haematological pathologies are especially vulnerable to infections in general, and to SARS-CoV-2 in particular. They also show a less favourable clinical evolution, with more serious cases and a higher death rate. In a cohort study carried out in two centres in Wuhan, China, by Wenjuan He et al. 34, they show 10% of COVID-19 incidence in haematological patients, which is much higher than in any other oncological patients, but it is similar to the rate observed in workers of the centre (7%). However, mortality differs in both groups: 62% in onco-haematological patients and 0% in workers, concluding that the incidence is similar in both groups, but it is much more serious in onco-haematological patients than in workers, which is what we have observed in our little study 35. In this sense, they state that an increase in mortality can be due to concomitant infections (similar to our study) in addition to the condition of immunosuppression. They conclude that this type of inpatients should be subsidiary of special isolation as well as special and rigorous clinical surveillance in the context of a pandemic.

Other investigations 36, 37, also in China, suggest a greater incidence of serious infection in patients with cancer than in patients without known neoplasia (39% vs 8%). Yu J et al. 37 suggest that frequent admissions to the Hospital and periodic checks, that are inherent to this type of patients, could therefore influence their susceptibility to getting COVID-19, due to a higher exposition risk.

Lastly, Malard et al., in a French study, observe almost 50% of incidence, with a non-favourable evolution in a high percentage 38. They conclude that patients with haematological neoplasia are more susceptible to COVID-19 and to experience a more severe disease and as a consequence, greater mortality (they estimate 40% per month). In the same way, they observe that in most of the patients a nosocomial origin is suspected and that they present at least one added comorbidity.

In conclusion, this nosocomial outbreak of SARS-CoV-2 in an Haematology-Internal Medicine Unit is likely to have its origins in the community environment, being the initial cases a healthcare provider and/or a companion who tested positive. However, the concurrence of multiple cases favoured its spread. Therefore, we considered that the following preventive and control measures in non-COVID-19 Units were necessary to avoid a nosocomial outbreak:

- Surveil and track every case due to the possibility of infections at least 48h before symptoms, both in patients and workers, and keeping an active follow-up both on inpatients and outpatients. With regard to workers, they should notify symptoms immediately to the Preventive Medicine Department as well as to self-isolate.
- Reduce generating aerosols by nebulizations or high-flow oxygen therapies, which can boost the spread of high viral loads in an environment that normally does not present an adequate ventilation. Replace them with equivalent therapies with the use of a closed chamber, if this is not possible, could increase the personal protection of workers.
- Clean thoroughly the designated areas for patients and professionals due to the possibility of viral particles left in the surfaces to return to the air or to favour the spread via contact.
- Control and verify the ventilation of the rooms and the connections between these, since they can facilitate the viral propagation (for instance, when opening windows), or perhaps assessing the temporary suspension of the ventilation.
- Close onco-haematological patients surveillance, which are more susceptible to get the disease and to present a most serious evolution due to their immunosuppression.

Results from this study has been taken into account in order to change the hospital policy. At this time, all patients are screened with PCR at admission and after 7 days (at 2 and 7 days after admission if baseline PCR was not available). In the presence of a confirmed case in a hospitalization ward, whether of health personnel, non-health personnel or a patient, all patients and workers of the ward are screened with PCR. Positives cases are isolated and the PCR screening is repeated after 5-7 days to all the personnel and patients. This is done successively until no positive is identified. At the present time, the rooms are more ventilated, no aerosols are generated, replacing them with cameras, and only one companion per patient wearing a mask is allowed. It should be noted the existence of greater PCR tests availability and higher traceability capacity at the present time.

## Figures and Tables

**Table 1:** Global characteristics of positive COVID-19 patients.

Patients	1	2	3	4	5	6	7	8	9	10
Age	79	67	88	78	62	73	77	73	69	63
Sex	F	F	F	F	M	M	M	M	M	M
HTA	Yes	No	Yes	No	Yes	Yes	No	Yes	Yes	Yes
Dyslipidemia	Yes	No	No	No	Yes	Yes	Yes	Yes	Yes	No
Diabetes	No	No	No	No	No	No	Yes	No	No	No
Obesity	No	No	Yes	No	No	No	No	No	Yes	No
Pulmonary disease	No	Yes	Yes	No	No	Yes	No	No	Yes	No
Renal disease	No	No	No	No	Yes	No	No	No	No	No
Diagnostic	Hematologic disease	Hematologic disease	Hematologic disease	Hematologic disease	Hematologic disease	Oncologic	Oncologic	Hematologic disease	Hematologic disease	Hematologic disease
Immunosuppressants	Yes	Yes	Yes	Yes	Yes	Yes	Yes	Yes	Yes	Yes
Status of his/her illness	Relapse	Progression	Not evaluated	Not evaluated	Very good partial response	Progression	Progression	Partial answer	Partial answer	Partial answer
Concomitant active infection	No	Yes	Yes	Yes	Yes	Yes	Yes	No	Yes	Yes
ECOG	0	4	4	4	3	4	4	2	3	1
Nebulizations / high flow O2	No	Yes	Yes	No	No	Yes	Yes	No	Yes	No
PCR-COVID -	No	No	20-03-202	No	No	28/03/2020	No	03/04/2020	10/03/2020 02/04/2020	25/03/2020 27/03/2020
PCR-COVID +	16/03/2020	19/03/2020	23/03/2020	31/03/2020	02/04/2020	03/04/2020	03/04/2020	05/03/2020	05/03/2020	06/04/2020
COVID severity	4	4	4	4	4	4	4	4	3	3
Death	No	Yes	Yes	Yes	No	Yes	Yes	No	No	No

ECOG: 0 Fully active, 1 Restricted in physically strenuous activity, 2 Capable of all self-care, unable to carry out any work activities, 3 Capable of only limited self-care, 4 Completely disabled, 5 Dead. Covid severity: 1 Asymptomatic, 2 Mild: Mild symptoms 3 Moderate: fever or respiratory symptoms, 4 Severe: saturation <93%, 5 Critical (ICU); HTA: Arterial hypertension, F: female, M: male

**Table 2:** Global characteristics of positive COVID-19 workers.

Workers	1	2	3	4	5	6	7	8	9	10	11	12
Age	32	47	34	34	29	47	57	62	28	45	42	48
Sex	M	M	F	F	F	F	F	F	M	F	F	F
HTA	No	No	No	No	No	No	Yes	No	No	No	No	No
Dyslipidemia	No	No	No	No	No	No	Yes	No	No	No	No	No
Diabetes	No	No	No	No	No	No	No	No	No	No	No	No
Obesity	Yes	No	No	No	No	No	No	No	No	No	Yes	No
COPD	No	No	No	No	No	No	No	No	No	No	Yes	No
Renal disease	No	No	No	No	No	No	No	No	No	No	No	No
PCR-COVID +	14/03/20	02/04/20	04/04/20	04/04/20	04/04/20	04704/20	05/04/20	06/04/20	06/04/20	07/04720	07/04/20	14/04/20
COVID severity	5	3	2	2	2	2	3	3	3	3	3	2
Death	No	No	No	No	No	No	No	No	No	No	No	No

Covid severity: 1 Asymptomatic, 2 Mild: Mild symptoms 3 Moderate: mild respiratory distress (saturation>90%), 4 Severe: saturation <90%, 5 Critical (ICU); HTA: Arterial hypertension; COPD: chronic obstructive pulmonary disease, F: female, M: male.
